# Legitimacy in the Repertoire of Contention: How Black Lives Matter Activists Justify Riots

**DOI:** 10.1007/s11133-025-09630-z

**Published:** 2026-03-17

**Authors:** Mathis Ebbinghaus

**Affiliations:** Department of Sociology, University of Oxford, 42–43 Park End Street, Oxford, UK

**Keywords:** Social movements, Riots, Repertoire of contention, Legitimacy, Black Lives Matter

## Abstract

Legitimacy is an overlooked precondition for a tactic’s availability within social movement repertoires. Drawing on semi-structured interviews with 37 Movement for Black Lives activists in Minneapolis and St. Paul, Minnesota, this article identifies a three-step process through which activists legitimize riots. First, activists reclassify riots as protest by lumping them with revered tactics, thereby splitting them from criminality. Second, activists engage in moral legitimation: they acknowledge the harm riots can cause to Black communities but frame them as justified counterviolence to state repression. Third, activists use instrumental legitimation. Despite potential reputational risks, they argue that riots impose costs on capitalism, delegitimize the state and lend credibility to subsequent nonviolent protests. By tracing how activists legitimate a controversial movement tactic, this article argues that legitimation work shapes tactical availability. This challenges views of the repertoire of contention as a fixed toolkit from which activists choose tactics they regard as strategically effective or aligned with their collective identities.

## Introduction

Social movement repertoires are commonly understood as sets of recognizable tactics that range from petitions and peaceful marches to acts of violent resistance. These tactics make social movements legible not only to external audiences and authorities, but also to activists themselves (Jasper 2006; McAdam 1982; McAdam et al. 2001; Tilly 2008). The dominant view holds that activists choose tactics based on political opportunities and their strategic potential to disrupt opponents, attract media attention, or extract concessions (Gamson 1975; Ganz 2009; McAdam 1983; Piven and Cloward 1979; Tarrow 1998; Wang and Soule 2016). Other strands of scholarship show that tactics also carry meaning: they express values and help forge collective identities that resonate within particular cultural contexts (Bernstein 1997; Bosco 2001; Cohen 1985; Melucci 1995; Pattillo-McCoy 1998; Taylor and Whittier 1995; Taylor et al. 2009; Touraine 1981 [1978]). More recently, scholars have examined the relational and discursive processes that drive tactical innovation (Gold 2022; Ring-Ramirez et al. 2014; Steinberg 1999), re-interpreted repertoires of contention through pragmatist action theory (Gross 2010), and highlighted how activists creatively recombine tactics to respond to shifting political opportunities (Gold 2022; Jansen 2017).

Social movements innovate or select tactics from their repertoire only after a prior step has occurred: legitimation, the process by which a movement tactic comes to be seen as acceptable, defensible, and appropriate for collective action. However, where legitimacy is made explicit in existing research, discussions typically concern external audiences – bystanders, media, or state actors – and focus on the question of when contentious acts are deemed justified in light of the perceived legitimacy or illegitimacy of their targets. For example, scholarship on public opinion shows that people interpret contentious action through shared cognitive templates that sort events into categories such as legitimate protest and illegitimate or criminal disruption, and that these classificatory processes shape judgments of protest legitimacy (Gøtzsche-Astrup and Gøtzsche-Astrup 2025; see also Schoon 2022). By contrast, far less attention has been devoted to how activists themselves make sense of which tactics to adopt. As Biggs (2013, 410) observes, “knowing about a successful tactic is not sufficient cause to adopt it […]. Potential protesters must consider it to be feasible, legitimate, and effective in their own circumstances.” My concern is with the second aspect, namely how activists’ beliefs about legitimacy are constructed so that a tactic becomes available within a social movement repertoire (Walker and Willer 2014).

Although legitimation of social movement tactics is often implicit, it becomes more directly apparent when we turn to one of the most controversial tactics in the wider repertoire of contention: riots. Defined as the “deliberate destruction of property and attacks on others” (Kawalerowicz and Biggs 2015, 675), rioting represents a particularly disruptive tactic in the social movement repertoire. Although the vast majority of activists engage in peaceful protest, nonviolent demonstrations are frequently accompanied by riots involving “looting, smashing windows, attacking cars, arson, and attacks on security personnel or particular targeted groups” (Goldstone 2012, 107). Precisely because of their visceral imagery and disruptive force, riots often provoke endorsement or condemnation from activists that help shape the climate in which violent protest is either encouraged or ostracized. Unlike peaceful marches or petitions, riots compel activists to explain why they are legitimate, thus offering insights into the otherwise hidden process of tactical legitimation. Yet what is missing is an account of movement-internal legitimation: how social movement activists come to regard specific tactics like riots as legitimate (Ring-Ramirez et al. 2014). Such an analysis complements existing studies on the role of external discursive opportunities in shaping tactical choices (Koopmans and Olzak 2004), and how bystanders negotiate the legitimacy of protest and repression in real time (Bloom 2022).

While long at the periphery of social movement scholarship (Seferiades and Hank 2012), riots are now firmly embedded in the repertoire of contention (Carter 2004; Case 2021, 2022; Clover 2016; Cobb 2014), motivating analyses of why riots occur (Kawalerowicz and Biggs 2015; McPhail 1971; Oberschall 1968; Olzak and Shanahan 1996; Olzak et al. 1996; Santoro and Broidy 2014) and how they influence political outcomes (Abbs and Gleditsch 2021; Enos et al. 2019; Heaney and Rojas 2014; Isemann et al. 2019; Jung et al. 2014; Setter and Nepstad 2022; Simpson et al. 2018; Wasow 2020). Yet despite the scholarly consensus that tactics shape social movement trajectories and successes (McAdam 1983; McCammon 2003; Morris 1993; Taylor and Van Dyke 2004; Taylor et al. 2009), there remain “painfully recurring debates” about the legitimacy of riots (Case 2022, 15). Existing exceptions either identify individual-level predictors – such as dissatisfaction with racial progress – for supporting militant tactics (Santoro and Fitzpatrick 2015) or offer rich ethnographic insights that center on the perspectives of participants (Dupuis-Déri 2013; Meckfessel 2016; Popovic and Miller 2015) as exemplified by Auyero’s analysis of Argentinian food rioters who morally differentiate between “justified” looting and “unjustified” theft (Auyero 2007). Missing from this literature is a general sociological model of how activists legitimize specific tactics within the social movement repertoire.

This article begins to fill that gap. Drawing on in-depth interviews with 37 committed Movement for Black Lives^1^ activists in Minneapolis and St. Paul and insights from participant observation, I reconstruct a three-step process through which activists legitimize riots. First, drawing on Zerubavel’s (1996) cognitive processes of classification, I identify a relational mechanism through which activists legitimize riots: they reclassify riots as legitimate protest by “lumping” them with revered episodes of resistance while simultaneously “splitting” them from criminality. Second, through boundary work (Lamont 2012), activists engage in moral legitimation by reframing riots as adequate acts of counterviolence against state repression despite concerns over the harm they can inflict upon Black communities. Third, activists turn to instrumental legitimation based on “ordinalization” (Fourcade 2016). Here, riots are ranked above other tactics due to their perceived effectiveness despite concerns over their reputational damage to the movement: riots impose costs on capitalism, delegitimize state authority, and lend greater credibility to nonviolent social movement tactics. Together, I argue that legitimation constitutes the overlooked precondition for a tactic’s availability within social movement repertoires.

The setting of Minneapolis–St. Paul, Minnesota, offers two distinct advantages for theorizing the legitimation of riots. First, debates over violent versus nonviolent resistance have continually animated struggles for Black liberation (Carmichael 1966; Malcolm 1964) and resurfaced in activist discourse during the George Floyd uprising of 2020 (Vortex Group 2023). Second, Minneapolis experienced significant riots during the 2020 protests following the killing of George Floyd, rendering activist discussions about the legitimacy of riots especially salient even among non-participants. By investigating activists who did not personally participate in riots, I was able to mitigate the notorious challenge of post-hoc rationalizations of violent behavior. Instead, I illuminate how tactics are legitimated within the broader social movement infrastructure at a core site of one of the largest protest waves in U.S. history.

The article proceeds as follows. The next section reviews scholarship on the repertoire of contention, highlighting how legitimacy has remained an implicit, yet undertheorized, dimension of tactical availability. I then outline the conceptual framework for the legitimation of riots, building on insights from Zerubavel (1996), Lamont (2012), and Fourcade (2016) and introduce the case. After describing data and methods, I present activists’ own accounts of how they legitimize riots as a movement tactic. The conclusion summarizes the article’s main contributions and briefly discusses its implications for future research.

## Legitimacy in the Repertoire of Contention

Charles Tilly argued that collective claim-making relies on a narrow set of recognizable tactics such as petitions, strikes, marches, riots, and the like that make social movements intelligible to audiences, authorities and activists themselves (Ebbinghaus 2024; Jasper 2006; McAdam 1982; McAdam et al. 2001; Tilly 2008). The repertoire of contention is durable: activists “cling to the same few forms of collective expression” and modify them only gradually, like jazz musicians improvising around standard tunes (Tilly 2006, xiii). Tilly distinguishes two analytic versions of the repertoire of contention. The weak version is simply a list of available tactics. The strong version links those tactics to “social relations, meanings, and actions” (Tilly 1993, 265), thereby directing attention to the political and cultural processes that render some tactics plausible and others unthinkable (Jansen 2017). Indeed, recent pragmatist scholarship now treats tactics in the repertoire of contention as a bundle of “affordances” that describe material and symbolic properties which shape what actors can do with them in situ (Gold 2022). Building on Rucht’s (2025) distinction between a broad reservoir of protest forms that exists in principle and a narrower subset that is culturally available to particular movements, I treat legitimation as the process through which activists render specific tactics acceptable and defensible within their moral and strategic universe, thereby enabling their incorporation into the movement’s operative repertoire.

Why, then, do activists choose one tactic over another within their social movement repertoire? Resource mobilization and political process theorists argue that social movements strategically pick tactics most effective at disrupting opponents, attracting media attention, and winning concessions from elites (e.g. Gamson 1975; Ganz 2009; Wang and Soule 2016); and that tactics evolve in response to political opportunities (McAdam 1983; Tarrow 1998). For example, “poor people’s movements” resorted to mass defiance when formal channels were closed (Piven and Cloward 1979), routine exposure to state violence habituated activists to escalate toward “gray-zone” confrontational tactics (Auyero 2007), and student movements lacking external allies adopted more confrontational tactics compared to those embedded in broader coalitions (Disi Pavlic 2020). By contrast, New Social Movements scholars stress that tactics also express values and collective identities (Bernstein 1997; Cohen 1985; Melucci 1995; Touraine 1981). Lesbian-feminist groups, for instance, organized non-hierarchical meetings to enact the egalitarian gender relations they sought (Taylor and Whittier 1995), while LGBTQ+ activists staged symbolic same-sex weddings that combined legal claims with community rituals (Taylor et al. 2009). When tactics resonate with community symbols – like call-and-response sermons or freedom songs in the Black Church (Pattillo-McCoy 1998) or vigils of grieving mothers that shielded protesters and shamed authorities (Bosco 2001) – tactics gain not just effectiveness but also legitimacy.

Framing theory describes how social movements package their tactics in language and symbols that resonate with widely held values and social practice (Benford and Snow 2000; Snow et al. 1986; Taylor et al. 2009). The nineteenth-century English cotton spinners, for example, justified their strikes through the prevailing idioms of political economy, liberal individualism, and even abolitionism, to frame their contentious actions as logical extensions of widely accepted principles (Steinberg 1999). This case illustrates how social movements either creatively *align* their tactics with familiar cultural frameworks or abandon tactics that they cannot so legitimize. It also shows that each tactic contains hidden possibilities for innovation (Gold 2022). When a familiar routine no longer works because of moral or pragmatic reasons, activists improvise and invent new tactical scripts (ibid.).

The neglect of legitimation processes becomes especially clear when we turn to the most controversial of movement tactics: riots. Riots typically invite public and media backlash for their collective violence (Diani 2012; Simiti 2012), earning them dismissive labels such as illegitimate, irrational, or even criminal. For a long time, social movement scholars, too, have characterized riots as eruptions outside of “real” contentious politics (Bracey 2016; Case 2021). Although it is certainly true that riots are rarely formally planned by social movement organizers, they have played central roles throughout uprisings. In recognition of this fact, McAdam (1983) describes the urban riot as a key tactic of the 1960 s movements in the United States precisely because they were understood within Black communities as political protests and helped trigger federal anti-poverty spending. For McAdam (1983, 750), riots represented “the last major tactical innovation” of that period; an innovation that brings the question of legitimation squarely into scholarship on social movement tactics.

The adoption of particular tactics depends not only on available resources, political opportunities, and collective identities, but also on whether activists deem a tactic to be legitimate (Biggs 2013). Following Tyler, I define legitimacy as the belief that a given action “is appropriate, proper, and just” in a way that generates a felt obligation to support or follow it (Tyler 2006, 375-400). Legitimation, in turn, refers to how such beliefs are produced and stabilized by embedding an action within a justificatory framework (ibid., 376). Legitimacy is thus not a property of tactics themselves but of their reception. As Schoon (2022) argues, legitimacy emerges from the relationship between an object and an audience, as structured by shared expectations and standards of evaluation. A tactic is legitimate when a relevant audience recognizes it as protest rather than deviance and accepts it as a fitting tactic in their repertoire of contention. This framework extends classical relational accounts of legitimacy (Johnson et al. 2006; Suchman 1995; Weber 1978) to the intra-movement level by treating activists themselves as the audience. Because movements address multiple, partly internal audiences (organizers, rank-and-file activists, local allies), legitimacy is audience-specific and internally contested rather than a matter of society-wide consensus. A tactic enters the movement’s operative repertoire only once these audiences regard it as legitimate. Building on this relational understanding (Emirbayer 1997), I theorize *tactical legitimation* as the process through which activists make contentious tactics acceptable inside the movement. This process unfolds through three analytically distinct but mutually reinforcing mechanisms: reclassification, moral legitimation, and instrumental legitimation. The next section develops a framework for tracing these mechanisms by focusing on the case of riots.

## Theorizing the Legitimacy of Riots

Drawing on different classification frameworks, I theorize tactical legitimation as a sequential relational process involving three distinct steps. Reclassification involves the cognitive shifting of boundaries whereby controversial actions become grouped (“lumped”) with legitimate protest activities and differentiated (“split”) from criminal behaviors (Zerubavel 1996). Moral legitimation entails framing contentious tactics as ethically defensible by linking them to widely accepted moral orders such as justice, self-defense, or equality (Boltanski and Thévenot 2006; Lamont 2012). Finally, instrumental legitimation involves pragmatic evaluations of tactics based on their perceived effectiveness or strategic payoffs, which are typically assessed through ordinal comparisons with other available tactics (Fourcade 2016).

*Reclassification –* Before collective actions as contentious as riots can be evaluated in terms of their legitimacy, they must first be rendered *classifiable* as political protest. Following Zerubavel (1991, 1996), classification entails cognitive boundary work through which actors “lump” diverse practices together under shared labels and “split” them from dissimilar ones. In Zerubavel’s (1996, 421) terms, social actors carve “islands of meaning” out of a continuous world by downplaying differences within categories and exaggerating the gaps between them. Through lumping, heterogeneous events such as street marches, sit-ins, and occupations become interchangeable variants of a single unit of meaning – say, “protest” – while splitting hardens the boundary that separates this cluster from adjacent ones such as “crime” or “disorder.” These classificatory moves are intersubjective: they are learned within particular thought communities and stabilized through language, organizational rules and routines, and spatial zoning that materializes mental distinctions. In this sense, classificatory schemes form a shared cognitive backdrop that organizes how members of a society perceive similarity, difference, and the meaningfulness of actions. Thus, legitimation of movement tactics depends on the categorical schemas available to audiences: when riots are grouped with canonical forms of protest (e.g., the Bastille, the Boston Tea Party, Stonewall) and distinguished from apolitical or criminal violence, they become recognizable as candidates for moral or instrumental evaluation as instances of contentious politics. Recent research on cultural models of contention elaborates this connection between classificatory schemas and legitimacy judgments, showing that public assumptions about what counts as legitimate and illegitimate tactics are structured by shared cultural models and display a high degree of stability (Gøtzsche-Astrup and Gøtzsche-Astrup 2025). Because disruptive tactics can reduce public support for a movement’s claims, activists face a strategic trade-off in deciding whether to deploy them (Wang and Piazza 2016). This trade-off, in turn, gives them incentives to engage in classificatory boundary work, so that disruptive tactics are recognised as protest and evaluated accordingly rather than treated as mere crime.

*Moral legitimation –* Once reclassified as legitimate protest, riots are justified morally through relational boundary-drawing between the counterviolence of the oppressed and the violence of authorities (Lamont 2012). Such framing resonates with Fanon’s *The Wretched of the Earth* where he (1968, 51) depicts insurgent violence as “a cleansing force” that frees the oppressed from fear and inferiority, thereby framing their violence as proportionate and righteous retaliation: *their* violence created the need for *our* violence. This logic is also echoed by civil rights leaders like Robert F. Williams (1998 [1962], 76) who argued: “When people say that they are opposed to Negroes “resorting to violence” what they really mean is that they are opposed to Negroes defending themselves and challenging the exclusive monopoly of violence practiced by white racists.” Activists also evoke moral legitimacy by connecting riots emotionally to community healing and collective honor. This emotive dimension of moral legitimation closely resembles participants’ justifications of their involvement that tend to highlight the “contentious effervescence” (Case 2021) of rioting and their affective value as expressions of collective healing in response to injustice (Kokoreff 2005). As one participant in the George Floyd uprising reflects: “Seeing a police precinct burn is a much-needed release for all those who have been forced inside one, for everyone who has been beaten inside it, for everyone who loves someone who has been murdered by the police. Seeing cops run scared from a righteous crowd is a release. It’s healing” (Kovich 2020, 138). Such testimonies illustrate the transformative impact of rioting on participants (Thompson 2010), which may also shape the moral legitimation of riots among activist audiences. By grounding violent tactics in higher principles like self-defense, liberation, or honor, activists invoke universalizable moral claims tied to the civic value of equality, the domestic value of protecting one’s own community, or the political value of freedom (Boltanski and Thévenot 2006).

*Instrumental legitimation* – Finally, instrumental legitimation involves ordinal judgments among tactics – “oriented to relative positions according to a stable rank-ordering criterion” (Fourcade 2016, 178) – whereby different forms of protest are ranked along a hierarchy of worth. Movements can make such rankings on several bases (moral resonance, expressive fit with movement values, aesthetic appeal), but a particularly salient basis in contentious politics is perceived strategic effectiveness. To legitimize riots instrumentally, then, activists can challenge common tactical rankings by emphasizing their relative effectiveness compared to widely accepted methods such as marches, vigils, or petitions. One strategy is to draw on historical examples; to claim, for instance, that urban riots hastened civil-rights legislation, that the Stonewall riots catalysed the gay liberation movement, or that the American Revolution is unimaginable without the Boston Tea Party. This line of argument hinges on the perceived affordances of riots in imposing costs and creating leverage in ways that other tactics cannot (Case 2022; Gold 2022). Instrumental legitimation thus makes use of arguments – circulating in public debate and developed in some strands of scholarship – that, under certain conditions, riots can yield political gains (Enos et al. 2019) even as these claims run against the broader literature emphasising the average effectiveness of disciplined nonviolent campaigns (Chenoweth and Stephan 2008, 2011; Gamson 1975). Episodes of the sort Badiou (2012, 16) calls “immediate riots” have been shown in some of this empirical work to impose direct economic costs, shape public perceptions, expose vulnerabilities in state control, and sometimes strengthen the bargaining power of subsequent nonviolent mobilization (Ketchley 2017; Leenders 2012; Tripp 2013).

While the legitimation process presented here unfolds sequentially – from reclassification to moral and instrumental legitimation – it is worth noting that these steps are nonlinear and mutually reinforcing. On one hand, initial classification of riots as political protests (rather than mere criminality) sets the stage for subsequent moral and instrumental justifications. This view is in line with classical sociological theory which argues that shared systems of classification underpin social life by structuring collective evaluations (Durkheim and Mauss 1963 [1903]); similarly, Zerubavel (1996) highlights that categorizing phenomena as similar or distinct constitutes a precondition for meaning making and evaluation. On the other hand, moral and instrumental legitimation themselves recursively shape the categorical boundary of what constitutes a “legitimate protest” versus what constitutes a “riot.” Classic texts underscore that evaluation constructs and solidifies categories: Becker’s (1963) labeling theory demonstrates that categories such as “deviance” emerge from moral judgments and social sanctions rather than anything intrinsic to the categories themselves. Thus, classification can be seen as both a prerequisite for, and an outcome of, moral and instrumental legitimation (Boltanski and Thévenot 2006).

Once riots are reclassified through lumping and splitting, activists combine moral claims which frame riots as responses to injustice derived from shared orders of worth (Boltanski and Thévenot 2006) with an ordinal logic that ranks riots alongside conventional tactics based on their perceived effectiveness.

## The Case: Black Lives Matter and the Minneapolis Riots

The Black Lives Matter movement stands in a long tradition of Black liberation struggles that have continually debated the moral and strategic legitimacy of violence in pursuit of justice (Garza 2014; Taylor 2016). From slave uprisings and Reconstruction-era revolts to the Civil Rights and Black Power movements, Black resistance has oscillated between appeals to nonviolence and calls for self-defense. The well-known contrast between Martin Luther King Jr.’s ethic of redemptive suffering and Malcolm X’s insistence on retaliatory self-protection epitomizes this dilemma. Whereas King advocated self-inflicted suffering (Vander Zanden 1963), Malcolm X asserted in his characteristically blunt manner “that if a four-legged or two-legged dog attacks a Negro he should be killed.” When major riots occurred during the George Floyd uprising of 2020, debates about the legitimacy of riots as a social movement tactic once again took center stage in local activist discourse.

### Research Site

Minneapolis and St. Paul, Minnesota, provide an analytically relevant setting for studying how activists conceptualize the legitimacy of rioting. The Twin Cities have experienced recurrent protests against police violence – from Ferguson in 2014 through the killings of Jamar Clark, Philando Castile, and Justine Damon – culminating in the George Floyd uprising of 2020 (Phelps et al. 2021). That uprising included the first and most destructive riots of the Movement for Black Lives, transforming Minneapolis into the symbolic center of a national debate over the legitimacy of violent protest. Because large-scale riots and sustained mobilization are deeply embedded in local activist memory, Minneapolis constitutes a critical case: if mechanisms of tactical legitimation were absent here, they would be unlikely to appear elsewhere. I thus sampled a site where debates about riots were particularly salient.

To contextualize the debates that followed, it is necessary to briefly recall how the events unfolded on the ground. Although peaceful demonstrations vastly outnumbered riots in Minneapolis and nationwide, between May 26 and June 1, 2020, the city witnessed some of the most high-profile unrest of the Black Lives Matter uprising: When protesters directed their anger at the 3^rd^ Precinct of Minneapolis and began climbing “over the fences that protected the precinct” (Vortex Group 2023, 18), police responded by firing rubber bullets. Protesters, in turn, threw rocks and water bottles at officers, at parked police SUVs, and at the building of the precinct itself. After Minneapolis mayor Jacob Frey “ordered police to evacuate […] the crowd ebulliently touched the building, live-streaming videos of the party-like atmosphere” (Navratil et al. 2020, in Phelps et al. 2021, 433). Once the police station caught fire, the crowd looked “upon these sublimed fires with awe and approval” (Vortex Group 2023, 22). By that time, looting and arson had already spread from the neighborhood around the 3^rd^ precinct to other parts of the city. The Minneapolis riots primarily targeted police stations, government buildings, and businesses. Business owners who pleaded to spare their lives’ work faced severe losses (The New York Times 2020). The nightclub where George Floyd once worked as a security guard was destroyed by arson (ibid.), and on Lake Street, small Black-owned businesses burned for over twelve hours (McEvoy 2020). The riots caused approximately $550 million in damage to roughly 1,500 properties (Rao and Meitrodt 2020), resulted in 604 arrests (Pham 2020), and at least two deaths were linked to the unrest (Jany 2020). Although the riots themselves lasted only briefly, they made a lasting impression on activists in Minneapolis and St. Paul, compelling them to ponder the legitimacy of riots as a tactic in their repertoire of contention (for an overview of the week’s chronology, see Phelps et al. 2021, 432-35).

## Data and Methods

### Data Collection and Analysis

This analysis forms part of a broader qualitative research project on protest motivations, activist trajectories, and tactics within the Movement for Black Lives. In the summer of 2022, I conducted in-depth interviews with 38 activists engaged in the Movement for Black Lives in Minneapolis and St. Paul. I specified my sample in three ways that are crucial for detailing the mechanisms through which activists conceptualize the legitimacy of riots. First, I clarified that none of my interviewees had personally participated in riots. Interviewing activists who did not personally participate is analytically advantageous because it minimizes post-hoc rationalizations of personal behavior. Instead, it directs attention to the social movement infrastructure in which riots are either encouraged or met with ostracism. In other words, this approach separates the actual tactics used by participants from the broader tactical repertoire that activists may consider legitimate (and thus may use in future episodes of contention). Second, to ensure that I interviewed committed social movement activists rather than movement allies or occasional protesters, I limited my sample to respondents who had been involved in social justice activism since at least the summer of 2020. Conducting interviews two years after the George Floyd uprising struck a balance between studying an established social movement repertoire and minimizing recall bias as emotionally intense episodes are typically remembered clearly (Barberena et al. 2014). Third, because my objective is to trace the mechanisms by which riots come to be seen as legitimate, I deliberately sampled on the dependent variable; that is, I interviewed activists who deem riots legitimate (Small 2009). Taken together, these criteria constitute what Mitchell (1983, 195) calls a crucial case: it is chosen for its theoretical leverage in revealing *how* riot legitimacy is constructed rather than to generalize descriptively to the broader population of Black Lives Matter activists.

Of the 37 activists who met the inclusion criteria for this analysis, 26 activists were Black, three identified as mixed-race, and eight were White; they were roughly balanced by gender. The overrepresentation of Black respondents likely reflects their prominence among committed Movement for Black Lives activists, though it could also result from snowball sampling or White activists’ reluctance to speak on behalf of the movement. For example, a few White interviewees canceled their interviews at the last minute, expressing discomfort about speaking “for” Black Lives Matter or the broader Movement for Black Lives. The White people who did agree to participate were deeply embedded within the activist networks of Minneapolis and St. Paul yet frequently prefaced their responses by acknowledging their positionality. For example, one interviewee explained: “As a White person I am probably not the best person to talk to, but […].” Interviews ranged from 40 minutes to three hours 30 minutes, with an average duration of 90 minutes.

I gained access to interviewees through snowball sampling. A local Black youth activist leader facilitated initial contact with the broader activist community. His leadership role connected him to activists across diverse age groups and with varying roles in the Movement for Black Lives. After I conducted the initial interviews, my respondents occasionally introduced me to additional participants; alternatively, I waited for potential interviewees to reach out to me directly. Proceeding in this way reduced barriers to participation and enhanced interpersonal trust. All participants were informed in advance about the general topics of our discussion and understood my role as a researcher. Interviews were held in person, except in five cases where logistical constraints made this difficult; in those instances, I conducted the interviews via Zoom. Despite the sensitive nature of the topics discussed, these Zoom interviews retained the depth and intimacy of the face-to-face encounters and confirmed findings from my in-person interviews.

At the beginning of each interview, I obtained informed consent from respondents to record the interview and to use the transcripts for research purposes. I started each interview by asking respondents about their pathway into social justice activism and the type of movement activities they were engaged in. The semi-structured format of the interviews allowed flexibility to introduce questions naturally, depending on how the conversations unfolded. It should be stressed that I did not begin any interview by explicitly asking about the role of violence in protest, as doing so would have been insensitive. Instead, I waited until the conversation organically progressed toward tactics – a topic typically raised by activists themselves due to its prominence in activist discourse rather than imposing an artificial narrative. Although I followed up when respondents introduced interesting ideas, I ensured that each interview fully covered the topics outlined in the interview guide. As a result, all interviews followed a similar structure and covered the same core topics. 

Throughout data collection I kept analytic memos (Hammersley and Atkinson 2007) to identify emerging themes. Rather than imposing my preconceived ideas, I adopted an iterative data collection process in which my curiosity was continuously updated through conversations and field observations on the ground – the term grounded theory is fitting. I visited activist sites such as George Floyd Square or the “Say Their Name” cemetery and attended local protests, film screenings by Black activist producers, and Black Street art. I began with open coding of early interviews and fieldnote sets to generate descriptive labels. I collapsed these labels into higher order categories like reclassification, moral legitimation, or instrumental legitimation, which then structured focused coding of the remaining corpus. The resulting analysis is inductive and based on the complete set of interviews and observations.

## Reclassifying Riots: Lumping and Splitting

Reclassification is the first category that emerged inductively from my data as the initial step of tactical legitimation. Across interviews, activists began by lumping riots into the broader category of protest, thereby implicitly or explicitly splitting them from opportunistic criminality. Public discourse typically classifies riots as criminal disorder, irrational violence, or apolitical chaos. Activists contested this stigmatized framing by redefining riots as collective acts of protest continuous with the long tradition of Black resistance. A 44-year-old Black interviewee at George Floyd Square described his support poetically: “Riots are love bombs. You know, riots are love, courageous love.” More prosaically another respondent stated: “Sometimes there is a time and a place to burn shit down.” Such endorsements were often grounded in personal histories of suffering, exemplified by Jones^2^, a 50-year-old Black activist:

Riot is the voice of the unheard. [….] It takes an immense amount of courage to actually […] say we cannot pretend that things are normal. We can’t swallow this line of bullshit anymore. What arises is a courageous act of truth-telling: the third precinct needed to be burnt down. When I was in high school, [the police] were kidnapping my friends, beating them up and leaving them down by the river.

The first step activists took to legitimize riots involved categorizing riots as a subtype of protest. Zerubavel terms this cognitive process “lumping.” As one Black organizer explained, protests and riots “come out of the same womb.” Cassandra, a 38-year-old Black feminist activist, similarly argued that any action aimed at disruption qualifies as protest:

Protest is there to disrupt business as usual. To say there is something wrong, then to scream it. So a riot is a kind of protest […], right, and I think most people forget that there are different ways to protest. Black people protest every day by breathing because this society does not want us alive. So every time I take a deep breath, I protest the society, I protest the society that does not value Black lives.

Respondents also enacted splitting by attributing the most destructive acts to outsiders – White supremacists or provocateurs – thus excluding those acts from the category of protest. Ron, a 48-year-old Black activist, recalled:

Me and my wife walked around in the neighborhood and saw the aftermath of the uprising. The tension […] – it is like eight o’clock in the morning. There were still people out there shouting. I called my friend [to tell that] the third precinct burnt down. And they all screamed – they just burnt down the precinct. And everyone was screaming: “Yeah! Yeah! Yeah!” And people were dancing.

As his account continued, Ron abruptly shifted direction:

At the same time […] Black businesses were being destroyed by White supremacists. White supremacists flew into town just to participate. It’s weird. I understand why they did this; this was an urban warfare zone. These right-wing militia groups, […] this is their dream come true. […] They are racists and they came into town to play a live-action game.

This senseless violence contrasted with the riots activists celebrated as emotionally charged acts of collective resistance, especially after other forms of protest had been exhausted. Malia, a Black woman who campaigns as a movement organizer alongside her husband, explained:

Black people in this country protest all the time. […] It’s not necessarily a collective protest but we complain on our jobs when we are being mistreated. We complain about the medical system when we’re not being given the care that we need. We complain that the education system is not educating our children. We protest. Part of being Black in America is to protest. […] So when it gets to the point of rioting that means that we've done everything that we can think of at that moment. The pressure has just gotten too high.

Together, these accounts illustrate how activists reclassify riots as legitimate by lumping them together with historically revered forms of protest, implicitly splitting them from criminality. Latent moral sympathies make this lumping move cognitively plausible. Only once riots are cognitively recategorized as protest can they be subjected to moral evaluation or instrumental comparison. This classificatory boundary-work therefore is the foundation for the subsequent steps of moral and instrumental legitimation.

## Moral Legitimation

Once activists reclassified riots as a legitimate form of protest through lumping and splitting, they next defended riots' moral legitimacy. In this section, I show how activists framed riots as justified counterviolence against structural oppression and state violence – an argument long made in Black radical thought (Davis 1971; Newton 1972). They further engaged in moral boundary-work by distinguishing righteous counterviolence from morally problematic violence that harmed Black community members (Lamont 2012).

### Counterviolence

Across interviews, activists described riots as morally justified counterviolence in response to enduring state-led violence against Black people. For example, a 38-year-old Black feminist who moved to Minneapolis and became deeply involved in organizing the local movement posed the following pointed question:

How can there be such an act of violence and then tell people not to be violent? Like that does not make sense. The human psyche can’t navigate that because the way people are wired is an eye for an eye. Like you take mine, I take yours. […] It’s like it is not okay for a society to use violence against Black bodies and then tell Black bodies not to be violent. That is not okay. If you want folks to stop being violent, then stop being violent.

The counterviolence of riots is morally justified because activists perceived it as far less severe than the state violence they had encountered. This perspective highlights what activists saw as societal hypocrisy: condemning Black-led violent protest while tolerating ongoing structural violence. Riots were thus portrayed as reactive, or as Philando lamented: “People were more concerned with who started the burning, instead of who started the fire. […] The constant fires. For every action is a reaction.”

One middle-aged Black man with a resonant voice detailed his teenage experience of humiliation and sexual assault at the hands of police. When our conversation turned to violent protest tactics, he expressed anger. Drawing on his own personal experience with state-led violence, he deemed it inappropriate to label riots as violent, offering insights into his rationale for conceptualizing riots as a morally justifiable form of counterviolence:

As for White America, they committed one of the most violent acts that you could ever, ever, ever imagine. How do you dare call me or anyone else using their voice “violent?” These White people shooting in the schools, these are White men. So how dare you say these Black Lives Matter activists are violent? You should see the violence. […] If those actions were not done, we wouldn’t be there.

Such emotionally charged testimonies illustrate how activists morally legitimized riots as actions that honored communal trauma and outrage. By framing riots as a justified reaction against violence, activists implicitly invoked broad moral frameworks – or “orders of worth” (Boltanski and Thévenot 2006) – that appealed to values of equality, self-defense, and liberation. Reflecting on America’s history, Philando and other activists like Muriel, a 25-year-old movement organizer, also drew upon the country’s violent past as historical justification for riots, weighing the violence inherent in rioting against the greater scale of historical violence:

America was built on violence. They took everything from people. They raped, they murdered, they gave people smallpox, they lynched. […] America has rioted from Day 1. Everything that they have done has been a riot. They have raped, they have tortured, they have raped, they have done all of this.

Similarly, Muriel, a 25-year-old activist, explained how she views riots as a justified response to the ongoing infringement on the rights of Black people in America. Her reflections, along with those previously provided, align closely with Paul Gilje’s observation that “to tell the story of rioting in American history is in large part to rehearse the story of *all* of American history” (Gilje 1996, 177).:

When our rights are under attack, what do we do? We fight back. It's okay, to kill black and brown bodies, but it’s not okay for us to fight back against that? We can do this the easy way, or we can do this the hard way.

Just as Black Block anarchists justified their militant anti-capitalism by arguing that state violence is “killing people every day,” casting state agents “the real butchers, […] whose hands are soaked in blood” (Dupuis-Déri 2013, 128), a consistent narrative emerged from my interview data: social movement activists first expressed that riots are morally justified counterviolence to far greater state violence.

### Morally Justified Despite Harming Black Communities

While activists justify riots as counterviolence against state violence, they also acknowledged that morally justified violence should not harm their own neighborhoods. Unlike some radical Black Power theorists like Assata Shakur (1988, 212) who argued that property destruction is justified when it targets non-Black owners, many of my respondents wrestled with this tension. A 24-year-old Black activist described efforts to mitigate those effects of the riots on Black-owned businesses like Gandhi Mahal Restaurant (Schwartz 2020), Hub Bike Co-op, and Midtown Global Market, a minority community hub (Bring Me The News 2020):

We do have elders […], disabled people […], and young people in the community who needed to benefit from these stores. […] But then you got people like me in the community who […] organized events and provided free foods and pop-up stores in Minneapolis. We were able to distribute resources to thousands of families in that time too. Those are the types of events that you didn’t see on the news.

By attributing the worst destruction to far-right outsiders, activists preserved the boundary between purposeful collective resistance and senseless destruction, thereby reinforcing both their earlier reclassification and their moral legitimacy. The crucial sociological point is not whether these suspicions were accurate (Sidner 2020), but that blaming outsiders allowed activists to avoid treating destruction as inherent to riots. In doing so, they could affirm riots as morally justified protests while absolving the movement of ethical responsibility for collateral harm. The shift from praising riots as righteous protest to blaming White-supremacist outsiders for the worst destruction illustrates activists' moral boundary-work (Lamont 2012). By locating the most destructive acts outside the movement, activists upheld a distinction between justified counterviolence (aimed at police or major chains) and unjustifiable harm to Black residents and small businesses. This moral boundary-work found a vivid illustration in the story of the so-called *Umbrella Man* who was filmed setting fire to Black-owned shops (Sidner 2020). Carlos recalled the scene:

We saw it. There was a man called “Umbrella Man”. He was with his umbrella. He had a special tool for setting things on fire. When you get people like that, see them vandalizing your communities with no license. Everything was being hit by any means necessary.

A moment later, Carlos pivoted from attributing violence to a known outsider to praising the riots. To morally legitimize riots despite the collateral damage to Black communities, activists thus engaged in a distinct form of boundary work. They externalized the most troubling violence by attributing it to “outside agitators.” The remaining harm inflicted by the movement itself can then be reframed as counterviolence against morally culpable targets, or if that was not possible, construed as a tragic but necessary cost of confronting state brutality; a stance documented in Auyero’s (2007) analysis of Argentine food riots. Although distancing oneself from particular harm is not the same as justifying it, activists' boundary work constrains the scope of legitimation by allowing activists to selectively legitimize certain riotous actions while condemning others. In contrast to my own findings with non-participants, anonymous riot participants in the George Floyd uprising insisted that riots were largely carried out by local Black residents. These participants dispute the “myth of the ‘outside agitator’” (Vortex Group 2023, 146) as a trope used “by conservatives and nonviolence champions alike to discredit militancy wherever it appears” (Osterweil 2019, 5). Comparing how these rival narratives circulate among participants and non-participants is a promising avenue for future research on tactical legitimation

## Instrumental Legitimation

Having morally justified riots, activists proceeded to the third and final step of legitimation: instrumental legitimation, which involves demonstrating that riots are effective. As theorized earlier, this third step involves an ordinal valuation (Fourcade 2016) through which activists rank riots relative to tactics like marches or petitions by emphasizing riots’ unique ability to impose costs, delegitimize the state, and strengthen nonviolent protest (Ketchley 2017; McAdam and Su 2002). Activists’ support for riots stemmed from the belief that violent disruption achieves outcomes beyond those possible with peaceful tactics alone. For example, a young Black activist explicitly related his support for riots to their capacity to trigger policy changes and legal action:

Rioting […] is a good thing because now you get people to pay attention to what’s going on. When rioting is happening, you start seeing laws getting passed, you start seeing police officers getting fired, and you start getting chiefs resigning. That’s what you see when riots start happening.

Daniel, a seasoned activist, viewed riots as valuable because they disrupt the status quo: “It’s all about disrupting the status quo, letting people know that we disagree, and we don’t want things to go on as normal, as business as usual.” More pointedly, Jackson, a 29-year-old activist of mixed-racial background, proclaimed: “If you burn down a precinct you can get what you want. If you do a demonstration where you're asking for permission and it's not dangerous, and it’s not violent, it’s a parade.”

Jackson’s reasoning echoes arguments in social movement scholarship (Cress and Snow 2000; Gamson 1975; Piven and Cloward 1966), which emphasize that disruptive tactics can trigger political responsiveness. Activists often accepted the success of riots at face value without evaluating whether similar outcomes might have been achieved in the absence of riots. Instead, personal experience or shared wisdom served as heuristic cues for evaluating the tactic’s effectiveness. For example, Michael, a 28-year-old White interviewee, explained how he learned from Black activists about the historical importance of riots:

That riots get things done is historical. And this is one of the things that I’ve learned at protests and speeches. Where they say that you know we can make as much noise as we want but only the riots bring the change we need.

The idea that riots serve as an effective tool for social and political change is also captured in the following statement, which connects the prosecution of the police officer who killed Daunte Wright on April 11, 2021, to the riots that erupted in response:

Part of me is, like, I wished the riots would have continued, and that there would be riots for all of these unjust murders. Because maybe we get justice then. I guess there has been when Daunte Wright was killed. So, this cop was prosecuted, and I think there was a little bit of rioting and looting after his murder too. So, like yeah, I think they go hand in hand. When there is this huge disruption when there is rioting, it sometimes does lead to prosecution and justice like with Daunte Wright.

Similarly, another activist attributed any positive outcome of the George Floyd uprising to the riots rather than to the peaceful protests that spread from Minneapolis to cities across the United States:

But the fact that we had riots is not a coincidence. The cop that killed [Floyd] was the first White cop that was prosecuted. I get frustrated when […] people who are not part of the movement say that the riots are so terrible.

Riots are recalled as catalysts for social and political change – events that made the “whole fucking world suddenly wanting to have conversations,” as one disability rights and Black Lives Matter activist put it. Peaceful protests, by contrast, were more frequently remembered in connection with “state violence, mass injuries, and people’s items being stolen and damaged.” One explanation for this sentiment is that riots tend to be more memorable than nonviolent protests and therefore more readily attributable to observable change.

### Costs to Capitalism

Despite expected heterogeneity in the salience of this theme (Small and Calarco 2022), many interviewees did not merely suggest that riots are effective tools for change but also elaborated on how riots inflict economic costs and challenge the property relations that are upheld by politicians. The anticapitalistic impetus of riots is presented as an indirect challenge to political power itself. One of my Black respondents elaborated:

We live in a capitalist system. It’s a capitalist country. By definition, money is the subject that has rights, it’s not people. […] The lawmakers are gonna listen when you fuck with their money. […] They are making laws for capitalism we have to take actions that attack their capital.

This rationale corresponds closely to arguments that the effectiveness of riots arises from their ability to impose immediate economic costs, thus pressuring political elites responsible for maintaining capitalist order. Another respondent argued that riots go beyond mere disruption – they are “shocking to people” – and thus pose a threat to politicians who are accountable to capitalist corporations:

It was very shocking to people who saw their buildings burn down. But I think the other thing is that it was harming systems and corporations. It [therefore] was a risk to politicians. There was physical pressure, […] I do think that when protests cause a disturbance to society that is when we will see change.

These illustrations focus on the costs of riots to capitalism, but they are also compatible with Guy Debord’s (1967) argument that looting represents an act of wealth redistribution. As one participant in the Minneapolis uprising wrote: “The looters were *directly abolishing property relations*, which are inextricable from the violence of anti-Blackness. Let us recall that the order of private property is what killed George Floyd in the first place. It is one thing to hold a sign that says, “Redistribute the Wealth”; it is another to decide that all that shit on the store shelves is ours for the taking – and take it” (Vortex Group 2023, 106).

### Delegitimation of the State

Another argument about the disruptive capacity of riots centers on their ability to provoke responses from the state. By creating disorder, riots reveal the state’s inability to fulfill its duties, thereby undermining the legitimacy of state institutions (Unity and Struggle 2022). A Black Lives Matter activist working for the Minnesota state legislature described this legitimation mechanism as follows:

[Riots are] helpful because there is no longer a discussion of the legitimacy of the law and what can happen when it is lost. […] But when there is an actual precinct burning right now, you can’t come outside [because of] a curfew, [when] there are national guards in the streets, the legitimacy of law enforcement is completely obliterated.

I followed up by asking my interviewee: “So, riots are a useful tool for change because they are disruptive?”

Yes, absolutely, in every sense of the word. All the time and that’s the tragedy, we must die, destroy, burn. […] It’s useful because the state recognizes this anarchy, if it doesn’t establish its own legitimacy, then the state can collapse.

This exchange illustrates the theoretical claim that riots expose the state’s vulnerability by challenging its fundamental claim to control violence and maintain order (Leenders 2012; Tripp 2013). In this view, the state can only curtail unrest and restore its own legitimacy by making concessions to rioters.

### Strengthening Nonviolent Protest

The third lesson is that activists treat the threat of riots as a strategic complement to nonviolent protest. In activists' accounts, riots function less as substitutes for peaceful mobilization than as a credible threat that increases the leverage of subsequent nonviolent protest. This mechanism is consistent with Ketchley's (2017, 19) argument that street-level mobilization during the early phase of the 2011 revolution in Egypt was “inextricably entwined with anti-regime violence,” including attacks on district police stations, which almost always culminated in the looting and arson of abandoned police stations (Ketchley 2017, 30). In my own interviews, one respondent argued that the potential for peaceful nonviolent protests to escalate into riots is what renders them threatening to policymakers:

A protest only works with the threat of a riot. If people don’t have a civil way of getting a response, they are just saying, this is not my government. [With the threat of a riot] they can blackmail people into government responding.

Against this backdrop, many activists referred to Malcolm X and Martin Luther King – the iconic figures of the Black liberation movement – as complementary rather than opposing voices in debates over violent versus nonviolent resistance. Activists viewed both leaders as mutually reinforcing elements within the broader struggle for Black liberation. As one young Black activist explained:

The reason that Martin Luther King was exalted above others by Whites is that he was nonviolent. [But] without Malcolm X there would be no Martin Luther King. Period. Because Malcolm X scared the White people. […] Malcolm X stirred the pot. If you didn’t have him, you wouldn’t have people listen to nonviolent protests.

These accounts echo what social movement scholars label the “radical flank effect” (Haines 1984; Jenkins and Eckert 1986; Ketchley 2017). It describes the notion that militant tactics can widen the range of concessions policymakers consider in response to their nonviolent counterpart by making the latter appear more moderate and credible. By highlighting this relational dynamic, activists’ legitimation of riots challenges simplistic dichotomies between violence and nonviolence in the repertoire of contention.

### Effective Despite Reputational Damage

Violent protests from 1960 to 1972 have been linked to negative media coverage, shifts in public perception, and eventually a “shift among Whites toward Republicans and tipp[ing] the election” (Wasow 2020, 638). Throughout our in-depth conversations, my interviewees openly acknowledged similar risks, expressing concern that protest violence might harm the movement’s image and erode support among movement audiences. Nevertheless, this consideration did not alter the perception that riots remain necessary because “if it were not for rioters, the media would probably pay no attention at all” (Osterweil 2019, 9). A 28-year-old White protester who regularly attends Black Lives Matter protests articulated this dilemma:

My preference is that there are fewer riots because […] I hate to see violence, and fire, and all that business getting harmed, residences getting harmed and people potentially getting harmed and the violent aura being attributed to the movement.

The quote illustrates the concern that protest violence could negatively affect public opinion of the movement, thereby dishonoring the “aura” of the movement and potentially threatening political successes. Similarly, Randall, a Black Jazz musician and performer at Black Lives Matter events, argued that violent protests were picked up by the media to discredit the movement:

The national media really looked at the uprising and, listen, everyone who protested was also considered a rioter. And that frustrated me. Yes, there is a difference. Even when it comes to rioting, I would say, it wasn’t my tool, it was more young people who were more into the chaotic stuff.

These statements speak to scholarly debates on protest tactics (Chenoweth and Stephan 2008, 2011; Wasow 2020). Activists recognize the risks of negative media portrayals and potential public backlash but remain convinced that without disruptive riots, the movement’s demands would go unnoticed. This aligns with large-N evidence that protest events that advance narrower claims are more likely to employ violent disruptive tactics, whereas broadly resonant claims are associated with disruptive but nonviolent tactics. This is consistent with the idea that activists calibrate tactical risk to the breadth of their potential support (Wang and Piazza 2016).

## Conclusion

Drawing on semi-structured interviews with 37 Movement for Black Lives activists, this article shifted the focus from viewing the “repertoire of contention” as a fixed toolkit, toward the relational process of legitimation that underpins it. Exploring how activists legitimize riots in a context where a riot ignited one of the largest racial justice movements in U.S. history, I show that legitimizing riots involves a three-step process. First, activists reclassify riots through lumping and splitting: riots are grouped within the category of “protest,” simultaneously splitting them from mere criminality. Second, activists engage in moral legitimation by redrawing symbolic boundaries to justify riotous destruction as morally justified counterviolence against state violence despite concerns over the harm they can inflict upon Black communities. Third, activists perform instrumental legitimation through one form of ordinalization: riots are ranked relative to marches, petitions, and other tactics in terms of their strategic utility despite concerns about reputational damage to the movement (Wasow 2020). This process of categorizing, moralizing, and ranking – all relational processes – sheds light on an overlooked aspect of the repertoire of contention: tactical legitimation*.*

This article did not offer an analysis of riot participation frames – which would invite post-hoc rationalization – but was concerned with tactical legitimation among non-participants. Nevertheless, the legitimacy of a controversial tactic can matter even in ostensibly nonviolent social movement contexts. For example, legitimacy enables moderate activists and organizations to accept riots on their “radical flank” to enhance the leverage of their peaceful actions (Haines 1984; Jenkins and Eckert 1986; Ketchley 2017). Once legitimated, a tactic also remains latently available for activation: Du Bois’s (2008 [1903]) concept of double consciousness illuminates this latency, as Black actors learn to live in a White-dominant order while judging it morally deficient. When state violence becomes unmistakable, this “second sight” converts dormant approval into defiance. This is not because of new principles but because an already legitimated commitment to resist suddenly finds a focal point for expression (Bloom 2022).

The process of reclassification, moral justification, and instrumental evaluation is sequential but operates both retrospectively and prospectively. In the aftermath of riots, activists engage in reactive legitimation as they reinterpret past events to render them politically and morally intelligible – a process akin to post-hoc rationalization. Yet these same justifications also act prospectively. By articulating moral and instrumental reasons for rioting, activists normalize disruptive contention and furnish a template for future action. Reactive legitimation thus lays the groundwork for proactive legitimation, as the meanings and justifications developed after a riot render such tactics available within the repertoire of contention. The Minneapolis riots of 2020 illustrate this dynamic, as post-hoc legitimation by activists helped re-embed riots as recognizable, if controversial, tactics within the discourse of the Movement for Black Lives. Similar dynamics appear in White’s (1989) study of IRA militants, who retrospectively framed their turn to violence as a justified and more effective response to repression. Whether the consonance between participant and nonparticipant rationales reflects prior legitimation or retrospective rationalization remains a question for future research. Future work might also marry the legitimation sequence identified here with Jansen’s (2017) account of situated innovation, asking whether only legitimated tactics get recombined into new repertoires and how delegitimation may curtail tactical innovation (Gold 2022).

The three-step legitimation model may have theoretical implications that travel beyond social movements insofar as they suggest a generalizable sequence through which controversial social practices become legitimized. This sequence begins with a classificatory act – defining the object of legitimation – followed by moral justification through appeals to shared moral orders and concludes with instrumental rationalization based on effectiveness. The sequential ordering of moral and instrumental legitimation resonates with broader sociological theories of the state. Habermas’s (1975) analysis of legitimation in late-capitalist bureaucracies shows that, as normative agreement weakens, states resort to performance and administrative output to secure compliance. Yet this output-oriented legitimation remains ultimately dependent on a prior normative basis and cannot fully replace it. My analysis of activist reasoning offers a micro-level parallel; activists tend to secure a moral foundation for a tactic before turning to arguments about effectiveness. In this sense, Habermas’s macro-level diagnosis of the continued dependence of legitimacy on normative validity finds an analogue in the sequential structure of tactical legitimation observed in this analysis.

Legitimation is the overlooked precondition for a tactic’s availability within social movement repertoires. Whether the tactic is a march, a boycott, or indeed a riot, activists must deem their tactics legitimate before they are available tools in the repertoire of contention. By showing how Movement for Black Lives activists perform this tactical legitimation, this article has centered the voices that speak – borrowing Martin Luther King Jr’s words – in the “language of the unheard.”

## Footnotes

1 I use the term *Movement for Black Lives* to describe the wider activist community from which I sampled my interviewees. This is because my interviewees are a diverse, decentralized network of people dedicated to racial justice, which included but was not limited to activists formally associated with Black Lives Matter. In contrast, to align my usage with prevailing academic and public discourse, I employ the term *Black Lives Matter* whenever I refer to the protest movement initiated in 2013 that rose to prominence during the protest wave of 2020.

2 I use pseudonyms throughout to ensure anonymity of my respondents.
